# Supplementary material for: Exome-wide analysis implicates rare protein-altering variants in human handedness
Source: Nat Commun. 2024 Apr 2;15:2632. doi: 10.1038/s41467-024-46277-w (PMC10987538; doi:10.1038/s41467-024-46277-w)
Supplement: Supplementary file 1 — Supplementary Information [file 41467_2024_46277_MOESM1_ESM.pdf]

Supplementary Information

Schijven et al., Exome-wide analysis implicates rare protein-altering variants in human handedness

Contents:

Pages 1-11: **Supplementary Tables 1-11**

Pages 12-16: **Supplementary Figures 1-5**

**Filtering step**	**Individuals remain**
Individuals in final exome release	469,804
Available and consistently reported variable across instances (handedness, country of birth, part of multiple birth)	469,316
Non-missing covariate data	460,149
Inlier in one of the genetic ancestry clusters	420,979
Consistent genetic and self-reported sex	420,707
Unrelated (at third degree) to other individuals in the dataset	357,825
**Individuals after filtering**	
Right-handed	*313,271*
Left-handed	*38,043*
Use both hands equally	*6,511*

Supplementary Table 1: Overview of consecutive sample-level filtering steps.

Chromosome	Blocks	All variants	Variants in WES target regions	Monoallelic variants removed	Variants pre-filtering	Variants fail average GQ	Variants fail missingness	Variants fail MAC	Variants fail AB	Variants removed in filtering	Variants post-filtering	TsTv pre-filtering	TsTv post-filtering	Multiallelic variants removed	Variants pre-analysis
1	97	2283839	1127481	10213	1117268	8391	11086	175891	148	184198	933070	2.53	2.64	145055	788015
2	71	1684050	809663	7196	802467	5121	6213	127867	117	132267	670200	2.39	2.48	103970	566230
3	56	1337316	657839	5694	652145	2692	3693	103721	118	107018	545127	2.46	2.57	83781	461346
4	39	921893	459462	4148	455314	2017	3196	72813	89	75562	379752	2.34	2.42	59721	320031
5	43	1017453	511565	4524	507041	1740	2422	80737	63	82911	424130	2.37	2.46	66605	357525
6	48	1139230	574832	5418	569414	3961	5804	90468	103	95028	474386	2.45	2.54	73885	400501
7	47	1085905	534597	5212	529385	6454	7950	82574	83	88160	441225	2.44	2.54	72505	368720
8	35	822199	408913	4104	404809	1896	2410	62377	57	64298	340511	2.37	2.46	58955	281556
9	42	976400	483862	4882	478980	4068	5290	74063	69	77654	401326	2.48	2.60	65557	335769
10	40	937587	453225	4094	449131	3457	4164	71154	77	74368	374763	2.47	2.56	58768	315995
11	57	1342372	686063	6620	679443	2465	2997	104203	105	106925	572518	2.52	2.63	92910	479608
12	52	1220874	575207	5341	569866	2741	3957	89159	80	92478	477388	2.54	2.65	72088	405300
13	18	410890	206429	2022	204407	534	750	32401	43	33124	171283	2.41	2.51	26527	144756
14	30	710187	360604	3459	357145	1608	2596	55871	46	58082	299063	2.50	2.61	47034	252029
15	34	791702	385601	3644	381957	4570	5546	60234	41	64065	317892	2.39	2.49	50843	267049
16	47	1073520	540142	6235	533907	5148	5895	78676	53	82499	451408	2.53	2.65	83838	367570
17	56	1320835	641064	6491	634573	3227	4230	96792	57	100082	534491	2.68	2.81	85549	448942
18	16	366766	183977	1842	182135	746	972	28597	27	29475	152660	2.45	2.53	24090	128570
19	65	1490050	782551	9464	773087	3478	4630	113453	75	117598	655489	2.68	2.81	114488	541001
20	25	578858	281470	2934	278536	908	1107	41906	33	42899	235637	2.71	2.84	38114	197523
21	11	243382	120677	1363	119314	1687	1892	18536	30	19584	99730	2.65	2.76	16254	83476
22	23	515225	254870	2753	252117	1867	2482	37103	17	38857	213260	2.83	2.98	35427	177833
X	24	573514	284530	1859	282671	4076	6186	76190	170	80339	202332	2.57	2.75	24794	177538
TOTAL	976	22844047	11324624	109512	11215112	72852	95468	1774786	1701	1847471	9367641	2.50	2.61	1500758	7866883

Supplementary Table 2. Variant quality control filtering and numbers of variants removed/remaining at each step. Abbreviations: WES: Whole-exome sequencing, GQ: Genotype quality, MAC: Minor Allele Count, AB: Allele Balance, TsTv: Transition-Transversion ratio.

Variant type	Putative impact	Note
chromosome_deletion	HIGH	Strict
chromosome_duplication	HIGH	Strict
chromosome_deletion	HIGH	Strict
exon_loss_variant	HIGH	Strict
exon_duplication	HIGH	Strict
exon_inversion	HIGH	Strict
frameshift_variant	HIGH	Strict
feature_ablation	HIGH	Strict
gene_fusion	HIGH	Strict
gene_fusion	HIGH	Strict
bidirectional_gene_fusion	HIGH	Strict
rearranged_at_DNA_level	HIGH	Strict
protein_protein_contact	HIGH	Strict
structural_interaction_variant	HIGH	Strict
rare_amino_acid_variant	HIGH	Strict
splice_acceptor_variant	HIGH	Strict
splice_donor_variant	HIGH	Strict
stop_lost	HIGH	Strict
start_lost	HIGH	Strict
stop_gained	HIGH	Strict
feature_ablation	HIGH	Strict
inframe_insertion	MODERATE	Strict, CADD>20. Broad, CADD>1.
disruptive_inframe_insertion	MODERATE	Strict, CADD>20. Broad, CADD>1.
inframe_deletion	MODERATE	Strict, CADD>20. Broad, CADD>1.
disruptive_inframe_deletion	MODERATE	Strict, CADD>20. Broad, CADD>1.
missense_variant	MODERATE	Strict, CADD>20. Broad, CADD>1.
splice_region_variant	MODERATE	Strict, CADD>20. Broad, CADD>1.
3_prime_UTR_truncation + exon_loss	MODERATE	Strict, CADD>20. Broad, CADD>1.
5_prime_UTR_truncation + exon_loss_variant	MODERATE	Strict, CADD>20. Broad, CADD>1.
sequence_feature + exon_loss_variant	MODERATE	Strict, CADD>20. Broad, CADD>1.
coding_sequence_variant	LOW	Excluded
initiator_codon_variant	LOW	Excluded
stop_retained_variant	LOW	Excluded
splice_region_variant	LOW	Excluded
splice_region_variant	LOW	Excluded
5_prime_UTR_premature_start_codon_gain_variant	LOW	Excluded
synonymous_variant	LOW	Excluded
start_retained	LOW	Excluded
stop_retained_variant	LOW	Excluded
coding_sequence_variant	MODIFIER	Broad, CADD>1.
downstream_gene_variant	MODIFIER	Broad, CADD>1.
exon_variant	MODIFIER	Broad, CADD>1.
gene_variant	MODIFIER	Broad, CADD>1.
duplication	MODIFIER	Broad, CADD>1.
intergenic_region	MODIFIER	Broad, CADD>1.
conserved_intergenic_variant	MODIFIER	Broad, CADD>1.
intragenic_variant	MODIFIER	Broad, CADD>1.
intron_variant	MODIFIER	Broad, CADD>1.
conserved_intron_variant	MODIFIER	Broad, CADD>1.
miRNA	MODIFIER	Broad, CADD>1.
transcript_variant	MODIFIER	Broad, CADD>1.
regulatory_region_variant	MODIFIER	Broad, CADD>1.
upstream_gene_variant	MODIFIER	Broad, CADD>1.
3_prime_UTR_variant	MODIFIER	Broad, CADD>1.
5_prime_UTR_variant	MODIFIER	Broad, CADD>1.

Supplementary Table 3. Functional annotations based on snpEff/Ensembl that were used to construct the 'strict' and 'broad' sets of variants (in addition to criteria based on canonical vs non-canonical transcripts and positions within genes - see main text). Combined Annotation Dependent Depletion (CADD) thresholds refer to phred-scaled CADD scores.

Strict variant set

Chr	Position	Gene	Ensembl ID	Effect	SE	P	Direction
9	137241374	*TUBB4B*	ENSG00000188229	1.07	0.22	9.9×10^{-7}	+-+?
2	85595771	*RNF181*	ENSG00000168894	0.66	0.16	2.2×10^{-5}	++-+
11	107504672	*ALKBH8*	ENSG00000137760	0.17	0.04	2.6×10^{-5}	++-+
6	10983789	*ELOVL2*	ENSG00000197977	0.59	0.14	2.7×10^{-5}	+---
5	119452583	*HSD17B4*	ENSG00000133835	0.17	0.041	4.3×10^{-5}	+++-
3	42091492	*TRAK1*	ENSG00000182606	0.14	0.034	5.8×10^{-5}	++++

Broad variant set

Chr	Position	Gene	Ensembl ID	Effect	SE	P	Direction
9	137241374	*TUBB4B*	ENSG00000188229	1.06	0.22	1.2×10^{-6}	+-+?
13	25249267	*MTMR6*	ENSG00000139505	0.18	0.045	3.8×10^{-5}	++++
11	107504672	*ALKBH8*	ENSG00000137760	0.13	0.032	6.9×10^{-5}	++-+
14	94037387	*OTUB2*	ENSG00000089723	-0.40	0.10	8.4×10^{-5}	--++
1	7920843	*TNFRSF9*	ENSG00000049249	0.22	0.055	9.2×10^{-5}	+++-

Supplementary Table 4. Genes showing rare-variant associations with left- versus right-handedness at nominal significance $P<1x10^{-5}$, based on the strict (top) and broad (bottom) variant annotation masks. Only *TUBB4B* remained significant after exome-wide multiple testing correction. Chr: chromosome. Position: gene start position on chromosome according to the GRCh38 reference human genome. Gene: gene symbol. Ensembl ID: Ensembl database gene identifier. Effect: association test beta effect size. SE: standard error of beta. P: nominal gene-based association P value. Direction: sign of beta in four ancestry groups (White, Asian, Black, Chinese; a question mark indicates that the test was not run due to insufficient numbers of variants in a particular group).

			Strict				Broad			
**Chr**	**Gene**	**Ensembl ID**	**Effect**	**SE**	**P**	**Direction**	**Effect**	**SE**	**P**	**Direction**
1	*ST3GAL3*	ENSG00000126091	0.013	0.093	0.89	+--?	−0.0030	0.061	0.96	+-+-
1	*VANGL2*	ENSG00000162738	0.080	0.053	0.13	+-++	0.083	0.053	0.11	+--+
1	*NME7*	ENSG00000143156	−0.23	0.087	8.0×10^{-3}	-++-	−0.12	0.064	6.7×10^{-2}	-+--
2	*FOXN2*	ENSG00000170802	0.33	0.12	6.7×10^{-3}	+++-	0.19	0.10	5.7×10^{-2}	+++-
2	*SH3RF3*	ENSG00000172985	0.0	0.025	1.0	+---	0.0018	0.022	0.94	+---
2	*ITGAV*	ENSG00000138448	0.051	0.072	0.47	++-+	−0.032	0.051	0.53	-+-+
2	*MAP2*	ENSG00000078018	3.1	2.3	0.18	+???	−0.0078	0.029	0.79	-+-+
3	*SATB1*	ENSG00000182568	0.12	0.076	0.10	+++-	0.094	0.071	0.18	+++-
3	*CNTN3*	ENSG00000113805	−0.014	0.043	0.75	--++	−0.0011	0.031	0.97	--+-
3	*ROBO2*	ENSG00000185008	*NA*	*NA*	*NA*	*NA*	−0.0081	0.036	0.82	+---
3	*RSRC1*	ENSG00000174891	−0.033	0.051	0.52	-+++	−0.063	0.050	0.21	-+++
4	*FAM13A*	ENSG00000138640	−0.0060	0.063	0.92	+---	−0.017	0.032	0.60	----
4	*SLC39A8*	ENSG00000138821	0.011	0.10	0.92	--+-	0.033	0.091	0.72	+-+-
5	*LINC02056*	ENSG00000248371	*NA*	*NA*	*NA*	*NA*	*NA*	*NA*	*NA*	*NA*
5	*TMEM161B-AS1*	ENSG00000247828	*NA*	*NA*	*NA*	*NA*	*NA*	*NA*	*NA*	*NA*
5	*TRIM36*	ENSG00000152503	0.086	0.088	0.33	++--	0.10	0.070	0.14	++--
6	*BPHL*	ENSG00000137274	0.044	0.091	0.63	++-+	0.036	0.084	0.67	++-+
6	*ABT1*	ENSG00000146109	0.076	0.11	0.49	++--	0.040	0.10	0.69	++--
6	*TUBB*	ENSG00000196230	0.66	0.55	0.23	+--?	0.66	0.55	0.23	+--?
6	*ECHDC1*	ENSG00000093144	0.076	0.12	0.52	+---	0.064	0.095	0.50	+-+-
7	*PAX4*	ENSG00000106331	−0.070	0.055	0.20	----	−0.011	0.040	0.79	--+-
8	*NDRG1*	ENSG00000104419	0.037	0.073	0.62	++++	0.038	0.070	0.59	++++
10	*BUB3*	ENSG00000154473	0.36	0.15	1.5×10^{-2}	+-+-	0.37	0.15	1.2×10^{-2}	+-+-
11	*SOX6*	ENSG00000110693	−1.1	3.2	0.73	-???	0.040	0.064	0.53	+-++
11	*NPAS4*	ENSG00000174576	0.064	0.088	0.47	+++-	0.037	0.030	0.21	++-+
11	*RSF1*	ENSG00000048649	0.0072	0.029	0.80	+++-	0.0074	0.026	0.78	+-+-
11	*CADM1*	ENSG00000182985	−0.037	0.058	0.53	-+--	−0.037	0.056	0.51	-+--
12	*TUBA1B*	ENSG00000123416	0.90	0.64	0.16	+???	0.90	0.64	0.16	+???
12	*ANKS1B*	ENSG00000185046	−1.1	1.3	0.37	-???	0.0040	0.056	0.94	+-+-
13	*WASF3*	ENSG00000132970	0.017	0.082	0.83	+---	6.0×10^{-4}	0.038	0.99	+---
14	*AL133166.1*	*NA*	*NA*	*NA*	*NA*	*NA*	*NA*	*NA*	*NA*	*NA*
14	*LINC00648*	ENSG00000259129	*NA*	*NA*	*NA*	*NA*	*NA*	*NA*	*NA*	*NA*
15	*FURIN*	ENSG00000140564	0.019	0.054	0.72	++--	7.0×10^{-4}	0.044	0.99	-+--
16	*ATXN2L*	ENSG00000168488	0.075	0.068	0.27	++++	0.051	0.056	0.37	++++
16	*SNTB2*	ENSG00000168807	−0.085	0.11	0.46	---+	−0.068	0.11	0.54	---+
16	*TUBB3*	ENSG00000258947	−0.048	0.22	0.82	-++-	0.052	0.21	0.80	-++-
17	*CRHR1*	ENSG00000120088	−0.052	0.10	0.60	-++-	−0.014	0.087	0.87	-+++
19	*TUBB4A*	ENSG00000104833	−0.11	0.17	0.51	--+?	0.024	0.058	0.67	+++-
19	*RABAC1*	ENSG00000105404	0.24	0.14	9.3×10^{-2}	+-++	0.16	0.14	0.23	+-++
22	*BCR*	ENSG00000186716	−0.017	0.028	0.55	----	−0.0062	0.026	0.81	+---
22	*TTC28*	ENSG00000100154	−0.0067	0.026	0.80	-+-+	−0.013	0.022	0.55	-+-+

Supplementary Table 5. Rare-variant association results from the present study, for 41 genes that were previously implicated in left-handedness by genome-wide association scanning based on common genetic variants (see main text). Results are shown for the strict and broad rare variant sets. Chr: chromosome. Gene: gene symbol. Ensembl ID: Ensembl database gene identifier. Effect: association test beta effect size. SE: standard error of beta. P: nominal gene-based association P value. Direction: sign of beta in four ancestry groups (White, Asian, Black, Chinese; a question mark indicates that the test was not run due to an absence of variants in a particular ancestry group). NA: gene has no canonical protein sequence and was not tested for association in the present study, or the gene-based test was run in none of the four ancestry groups.

Continuous variable	Effect size (general linear model)	SE	t	P
Speech-Reception-Threshold: Left ear	0.24	0.30	0.79	0.43
Speech-Reception-Threshold: Right ear	6.0×10^{-3}	0.30	0.020	0.98
Visual acuity: Left eye	-8.9×10^{-3}	0.036	-0.25	0.81
Visual acuity: Right eye	9.9×10^{-4}	0.035	0.03	0.98
**Categorical variable**	**Effect size (binomial regression)**	**SE**	**z**	**P**
Hearing difficulties/problems	0.23	0.20	1.13	0.26
Hearing aid user	0.38	0.42	0.90	0.37
Eye problems/disorders	-0.10	0.33	-0.29	0.77
Glasses or contact lenses	-0.09	0.31	-0.30	0.77

Supplementary Table 6. No significant associations between *TUBB4B* variant carrier status and vision or hearing problems in the UK Biobank. SE: Standard error of effect size (from general linear regression for continuous variables and binomial regression for binary variables). P: nominal P value from two-tailed testing.

			Strict				Broad			
**Chr**	**Gene**	**Ensembl ID**	**Effect**	**SE**	**P**	**Direction**	**Effect**	**SE**	**P**	**Direction**
1	*POGZ*	ENSG00000143442	0.11	0.076	0.13	++--	0.052	0.048	0.28	+++-
1	*ASH1L*	ENSG00000116539	-7.0×10^{-4}	0.037	0.99	-++-	−0.0045	0.032	0.89	-+--
2	*SCN2A*	ENSG00000136531	1.0×10^{-4}	0.039	1.0	+---	−0.0031	0.038	0.93	+---
3	*SLC6A1*	ENSG00000157103	−0.14	0.14	0.34	----	−0.028	0.049	0.57	----
3	*CTNNB1*	ENSG00000168036	−0.16	0.12	0.17	----	−0.057	0.073	0.44	--+-
3	*FOXP1*	ENSG00000114861	0.13	0.081	0.11	++--	0.17	0.046	2.3×10^{-4}	++++
4	*ANK2*	ENSG00000145362	−0.022	0.024	0.37	----	−0.028	0.018	0.11	----
6	*SYNGAP1*	ENSG00000197283	−0.066	0.085	0.44	--+-	−0.037	0.075	0.62	-++-
6	*ARID1B*	ENSG00000049618	0.013	0.030	0.67	+-+-	0.0034	0.028	0.90	+-+-
7	*GIGYF1*	ENSG00000146830	−0.18	0.69	0.79	---?	0.025	0.046	0.60	+-+-
10	*PTEN*	ENSG00000171862	−0.11	0.21	0.61	--++	−0.13	0.19	0.52	---+
11	*DEAF1*	ENSG00000177030	−0.045	0.080	0.57	---+	0.029	0.037	0.44	+--+
11	*KMT5B*	ENSG00000110066	−0.10	0.10	0.32	--+?	−0.082	0.040	4.2×10^{-2}	-++-
12	*GRIN2B*	ENSG00000273079	0.019	0.085	0.83	+-+-	−0.047	0.076	0.54	----
12	*MED13L*	ENSG00000123066	0.017	0.037	0.64	+---	0.026	0.028	0.36	+---
14	*CHD8*	ENSG00000100888	0.0096	0.036	0.79	+++-	−0.0033	0.032	0.92	-+--
15	*CHD2*	ENSG00000173575	−0.13	0.053	1.7×10^{-2}	-+--	−0.12	0.042	5.3×10^{-3}	----
16	*ANKRD11*	ENSG00000167522	0.032	0.038	0.41	+++-	0.014	0.024	0.55	+++-
17	*KDM6B*	ENSG00000132510	−0.025	0.038	0.50	--+-	−0.033	0.029	0.24	---+
17	*TLK2*	ENSG00000146872	0.014	0.17	0.93	-++-	−0.0048	0.16	0.98	-++-
20	*ADNP*	ENSG00000101126	0.034	0.079	0.66	+---	0.020	0.044	0.66	++--
21	*DYRK1A*	ENSG00000157540	0.010	0.068	0.88	++--	0.012	0.065	0.86	++++
21	*DSCAM*	ENSG00000171587	0.17	0.047	3.6×10^{-4}	++--	0.15	0.044	5.5×10^{-4}	++--
22	*SHANK3*	ENSG00000251322	*NA*	*NA*	*NA*	*NA*	0.040	0.038	0.29	+---

Supplementary Table 7. Rare-variant association results from the present study of left-handedness, for 24 genes that were previously implicated in autism by large-scale exomic rare-variant association (see main text). Results are shown for the strict and broad rare variant sets. Chr: chromosome. Gene: gene symbol. Ensembl ID: Ensembl database gene identifier. Effect: association test beta effect size. SE: standard error of beta. P: nominal gene-based association P value. Direction: sign of beta in four ancestry groups (White, Asian, Black, Chinese; a question mark indicates that the test was not run due to an absence of variants in a particular ancestry group). NA indicates that the gene-based test was run in none of the four ancestry groups for a particular gene.

			Strict				Broad			
Chr	Gene	Ensembl ID	Effect	SE	P	Direction	Effect	SE	P	Direction
5	*TRIO*	ENSG00000038382	−0.12	0.038	2.1×10^{-3}	--+-	−0.072	0.028	1.1×10^{-2}	--+-
7	*SP4*	ENSG00000105866	−0.078	0.079	0.32	-+--	−0.070	0.060	0.24	-+-+
7	*CUL1*	ENSG00000055130	0.41	0.17	1.4×10^{-2}	+-+-	0.34	0.18	6.1×10^{-2}	+-+-
8	*XPO7*	ENSG00000130227	0.065	0.11	0.57	++--	0.057	0.088	0.51	++--
8	*RB1CC1*	ENSG00000023287	0.0068	0.061	0.91	+-+-	−0.040	0.033	0.23	--+-
15	*HERC1*	ENSG00000103657	−0.015	0.026	0.55	-+-+	−0.0091	0.022	0.68	-+-+
16	*GRIN2A*	ENSG00000183454	0.0016	0.038	0.97	+---	−0.016	0.027	0.54	----
16	*SETD1A*	ENSG00000099381	−0.021	0.045	0.64	----	0.0082	0.035	0.81	+-+-
17	*CACNA1G*	ENSG00000006283	−0.034	0.041	0.41	-+-+	−0.019	0.034	0.57	-+-+
23	*GRIA3*	ENSG00000125675	-7.0×10^{-4}	0.14	1.0	-++-	0.052	0.12	0.66	+-+-

Supplementary Table 8. Rare-variant association results from the present study of left-handedness, for 10 genes that were previously implicated in schizophrenia by large-scale exomic rare-variant association (see main text). Results are shown for the strict and broad rare variant sets. Chr: chromosome. Gene: gene symbol. Ensembl ID: Ensembl database gene identifier. Effect: association test beta effect size. SE: standard error of beta. P: nominal gene-based association P value. Direction: sign of beta in four ancestry groups (White, Asian, Black, Chinese).

Chr	Gene	Ensembl ID	Strict				Broad			
			Effect	SE	P	Direction	Effect	SE	P	Direction
1	*GBA1*	ENSG00000177628	-0.0011	0.0386	0.9764	++--	0.0005	0.0363	0.9889	++--
2	*CAPN10*	ENSG00000142330	-0.0195	0.0261	0.4541	-+--	-0.0183	0.0248	0.4613	-+--
12	*LRRK2*	ENSG00000188906	0.0307	0.0282	0.2758	+-++	0.0293	0.0259	0.2572	+--+
19	*B3GNT3*	ENSG00000179913	0.0059	0.0497	0.9051	+--?	0.0169	0.0416	0.6854	++++

Supplementary Table 9. Rare-variant association results from the present study of left-handedness, for 4 genes that were previously implicated in Parkinson's disease large-scale exomic rare-variant association (see main text). Results are shown for the strict and broad rare variant sets. Chr: chromosome. Gene: gene symbol. Ensembl ID: Ensembl database gene identifier. Effect: association test beta effect size. SE: standard error of beta. P: nominal gene-based association P value. Direction: sign of beta in four ancestry groups (White, Asian, Black, Chinese).

Chr	Gene	Ensembl ID	Strict				Broad			
			Effect	SE	P	Direction	Effect	SE	P	Direction
6	*TREM2*	ENSG00000095970	-0.0749	0.122	0.5395	-+--	-0.068	0.0355	0.05552	-++-
9	*ABCA1*	ENSG00000165029	0.0174	0.0289	0.5477	+++-	0.0244	0.0243	0.3158	+-+-
11	*SORL1*	ENSG00000137642	0.0113	0.0312	0.7166	++--	0.0031	0.0266	0.9073	++-+
15	*ATP8B4*	ENSG00000104043	0.0117	0.0345	0.7354	+-+-	0.0051	0.0273	0.8518	+-+-
19	*ABCA7*	ENSG00000064687	0.0185	0.0299	0.5354	+--+	0.0214	0.0253	0.3962	+--+

Supplementary Table 10. Rare-variant association results from the present study of left-handedness, for 5 genes that were previously implicated in Alzheimer's disease by large-scale exomic rare-variant association (see main text). Results are shown for the strict and broad rare variant sets. Chr: chromosome. Gene: gene symbol. Ensembl ID: Ensembl database gene identifier. Effect: association test beta effect size. SE: standard error of beta. P: nominal gene-based association P value. Direction: sign of beta in four ancestry groups (White, Asian, Black, Chinese).

		Excluded	Included	Excluded (%)	Included (%)
**Handedness**	Right	103689	313271	93.6%	87.5%
	Left	5705	38043	5.2%	10.6%
	Both hands equally	1361	6511	1.2%	1.8%
**Sex**	Female	62635	191802	56.2%	53.6%
	Male	48856	166023	43.8%	46.4%
**Country of birth**	England	80295	284848	72.9%	79.6%
	Wales	4322	16437	3.9%	4.6%
	Scotland	7602	30196	6.9%	8.4%
	Northern Ireland	527	2360	0.5%	0.7%
	Republic of Ireland	1006	3621	0.9%	1.0%
	Elsewhere	16464	20363	14.9%	5.7%
**Ancestry cluster**	White	68197	343781	61.2%	96.1%
	Asian	845	7052	0.8%	2.0%
	Black	579	5729	0.5%	1.6%
	Chinese	96	1263	0.1%	0.4%
	Not clustered / mixed cluster	41774	0	37.5%	0.0%
**Part of multiple birth**	No	100980	350039	97.7%	97.8%
	Yes	2400	7786	2.3%	2.2%
**Exome sequencing batch**	First 50k	11362	38310	10.2%	10.7%
	All other	100129	319515	89.8%	89.3%
**Year of Birth (Mean, SD)**	-	1952 (8)	1951 (8)	-	-

Supplementary Table 11. Information on handedness and other variables that were used as covariates or for stratifying the analysis, in individuals excluded during sample-level filtering, versus those remaining after sample-level filtering.

Supplementary Figures

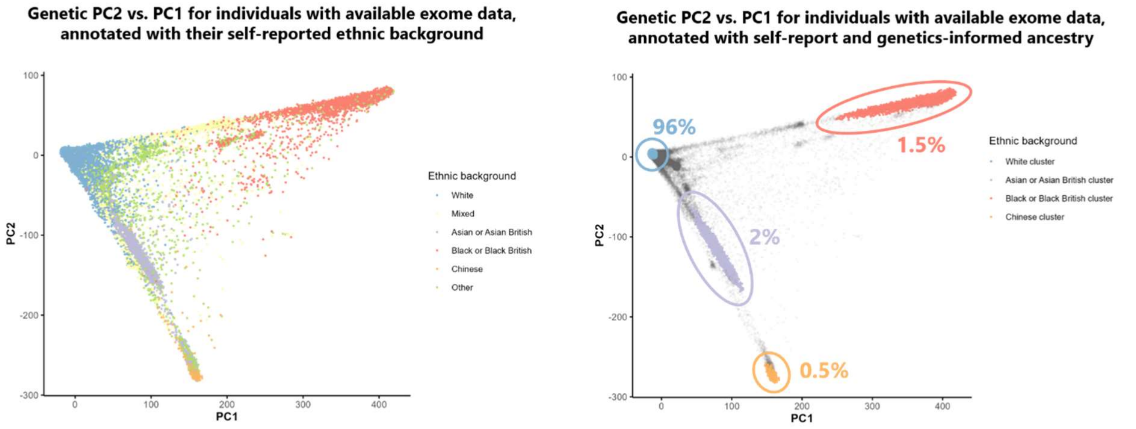

Supplementary Figure 1. Illustration of the process for defining genetic data-driven clusters. The goal was to define relatively genetically homogeneous groups, to avoid bias in genetic association analysis with handedness. Left panel: Individuals plotted according to the first principal component (PC1) and second principal component (PC2) that capture genome-wide diversity, and coloured according to their self-reported ethnicities. Right panel: Again, the individuals are plotted according to PC1 and PC2, but are now coloured according to Bayesian clustering within each self-reported group separately. Individuals marked grey in the right panel were not assigned to any clusters indicated in that panel, and were excluded from genetic association analysis with handedness. The illustration is given with respect to PC1 and PC2, but clustering was also performed on PC3 together with PC4, and PC5 together with PC6 (see main text). The approximate percentages of individuals within each are indicated (the exact numbers used for analysis are given in Table 1 of the main text).

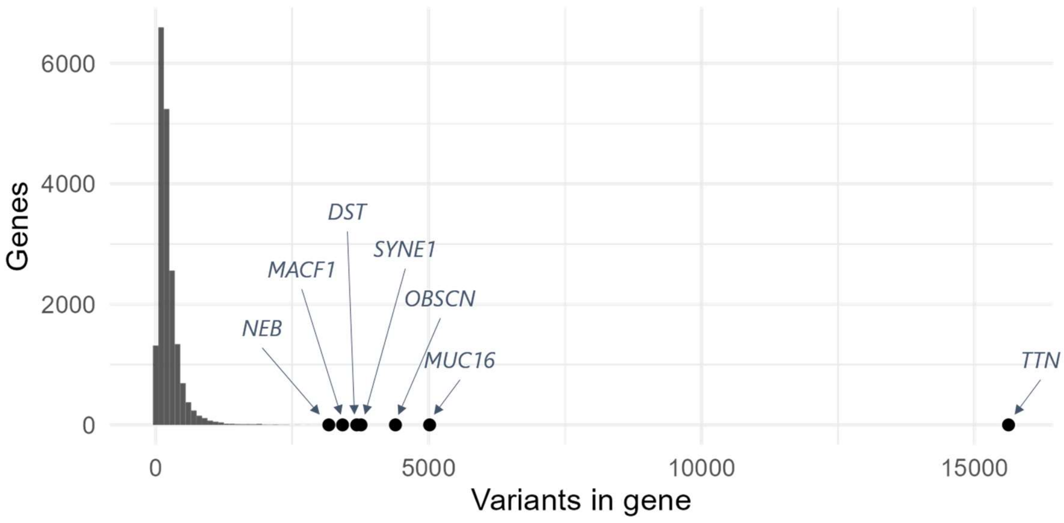

Supplementary Figure 2. Numbers of variants per gene with frequencies ≤ 1% after quality control filtering. 114 genes contained less than 10 variants. The gene *TTN* (titin) would not run through gene-based association testing in the White (largest) ancestry group, since its exceptionally high number of variants presented a computational problem. This gene encodes a large abundant protein of striated muscle.

Strict variant mask (62 variants)

Ancestry	N Right	N Left	Beta	P-value
white	300435	37177	1.118	4.7e-06
asian	6421	433	-1.143	0.53
black	5237	381	0.606	0.64
**Meta-analysis**	**312093**	**37991**	**1.071**	**9.9e-07**

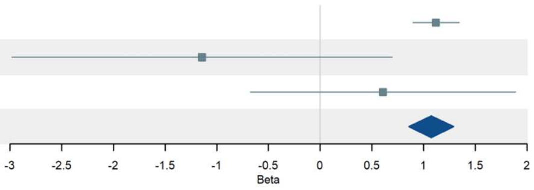

Broad variant mask (63 variants)

Ancestry	N Right	N Left	Beta	P-value
white	300435	37177	1.107	5.7e-06
asian	6421	433	-1.143	0.53
black	5237	381	0.606	0.64
**Meta-analysis**	**312093**	**37991**	**1.06**	**1.2e-06**

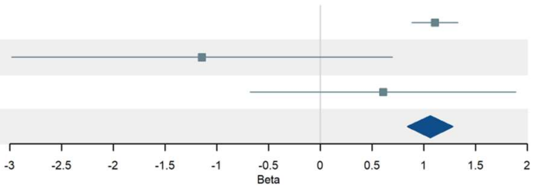

Supplementary Figure 3. *TUBB4B* rare-variant associations with left- versus right-handedness across ancestry groups. (No *TUBB4B* variants meeting the strict or broad criteria were found in the Chinese group, which was therefore not included in gene-based meta-analysis for *TUBB4B*). For the separate ancestry groups, the squares indicate the beta (effect size) values, with the lines indicating the standard errors. For the meta-analyzed effect, the diamond is centered on the beta value and its width indicates the standard error.

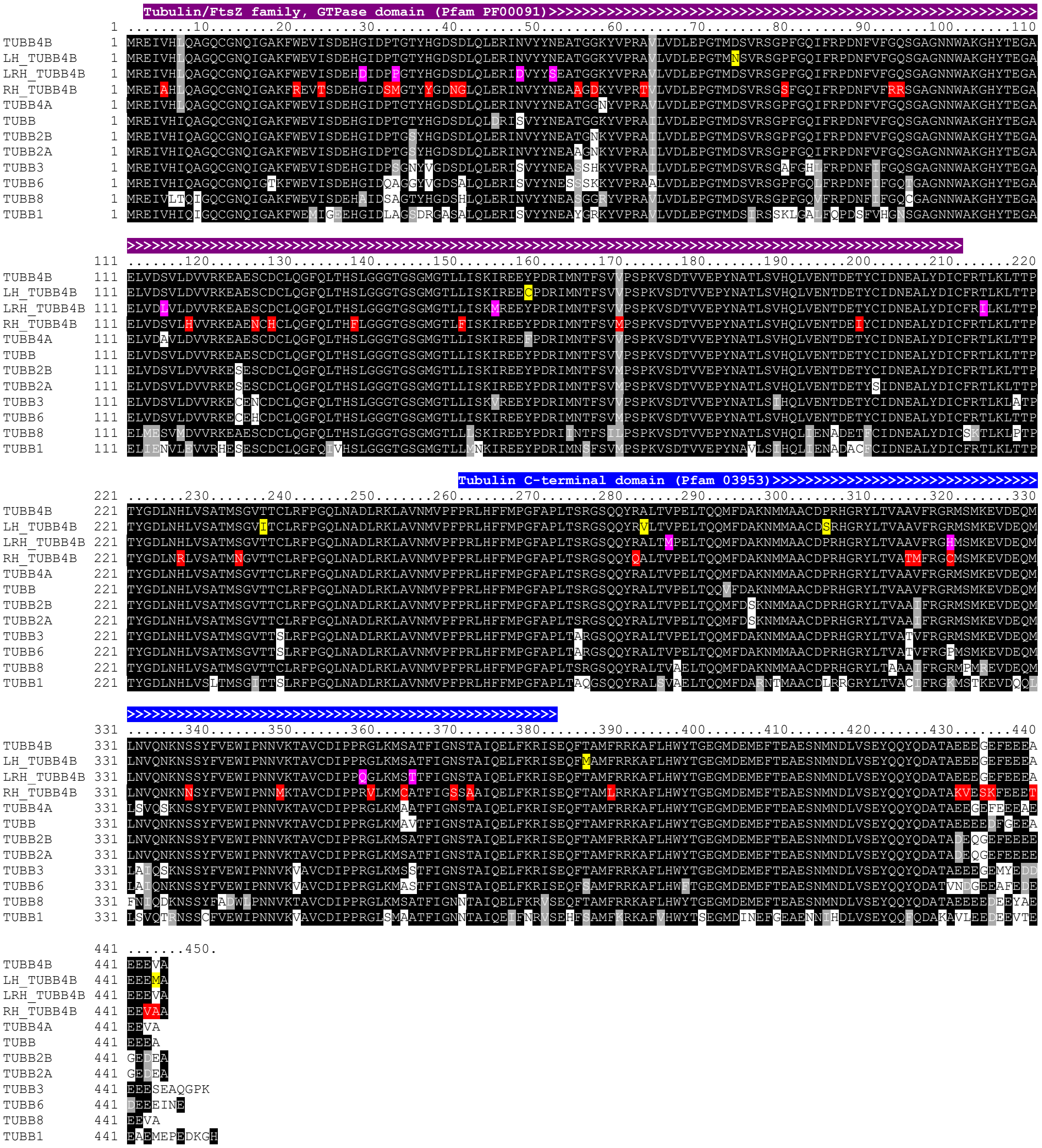

Supplementary Figure 4. Amino acid changes caused by rare *TUBB4B* missense variants in the UK Biobank that met the 'strict' functional annotation for having deleterious effects (see main manuscript). The canonical TUBB4B protein sequence is shown in the top black-highlighted line of each panel. The line 'LH_TUBB4B' shows variants found only in left-handers and they are highlighted in yellow. The line 'LRH_TUBB4B' shows variants found in both left-handers and right-handers, and they are highlighted in pink. The line 'RH_TUBB4B' shows variants found only in right-handers and they are highlighted in red. Some variants occurred in more than one individual (see Figure 2 of the main manuscript). The lower lines show the canonical protein sequences of all other human beta tubulin paralog genes, to understand how variable each site can be across human paralogs. Shading indicates similarity of residues across isotypes. Purple and blue colours indicate protein domains.
Note that human TUBB4B protein sequence conservation with its orthologs across vertebrate species (not shown in the figure) is extremely high: 100% amino acid identity conservation in *Pan troglodytes, Macaca mulatta, Mus musculus, Bos taurus, Canis lupus familiaris*; 99.78% conservation in *Rattus norvegicus*; 99.55% conservation in *Gallus gallus*; 99.33% conservation in *Xenopus tropicalis*.

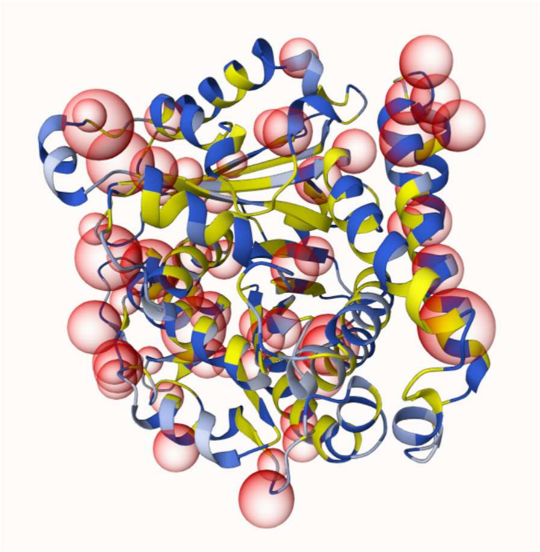

Supplementary Figure 5. Locations of the 60 rare TUBB4B missense variants in the UK Biobank that met the 'strict' functional annotation for having deleterious effects (see main manuscript), visualized against the three-dimensional structure of the canonical protein. Red circles indicate the locations of the missense variants. Blue and yellow indicate hydrophobicity of the canonical amino acids (blue lower, yellow higher).
