# Supplementary material for: Exome-wide analysis implicates rare protein-altering variants in human handedness
Source: Nat Commun. 2024 Apr 2;15:2632. doi: 10.1038/s41467-024-46277-w (PMC10987538; doi:10.1038/s41467-024-46277-w)
Supplement: Supplementary file 2 — Reporting Summary [file 41467_2024_46277_MOESM2_ESM.pdf]

nature portfolio

Corresponding author(s): Clyde Francks

Last updated by author(s): Feb 8, 2024



# Reporting Summary

Nature Portfolio wishes to improve the reproducibility of the work that we publish. This form provides structure for consistency and transparency in reporting. For further information on Nature Portfolio policies, see our Editorial Policies and the Editorial Policy Checklist.

## Statistics

For all statistical analyses, confirm that the following items are present in the figure legend, table legend, main text, or Methods section.

| n/a | Confirmed | |
|---|---|---|
| ☐ | ☒ | The exact sample size (*n*) for each experimental group/condition, given as a discrete number and unit of measurement |
| ☐ | ☒ | A statement on whether measurements were taken from distinct samples or whether the same sample was measured repeatedly |
| ☐ | ☒ | The statistical test(s) used AND whether they are one- or two-sided<br>*Only common tests should be described solely by name; describe more complex techniques in the Methods section.* |
| ☐ | ☒ | A description of all covariates tested |
| ☐ | ☒ | A description of any assumptions or corrections, such as tests of normality and adjustment for multiple comparisons |
| ☐ | ☒ | A full description of the statistical parameters including central tendency (e.g. means) or other basic estimates (e.g. regression coefficient) AND variation (e.g. standard deviation) or associated estimates of uncertainty (e.g. confidence intervals) |
| ☐ | ☒ | For null hypothesis testing, the test statistic (e.g. *F*, *t*, *r*) with confidence intervals, effect sizes, degrees of freedom and *P* value noted<br>*Give P values as exact values whenever suitable.* |
| ☒ | ☐ | For Bayesian analysis, information on the choice of priors and Markov chain Monte Carlo settings |
| ☒ | ☐ | For hierarchical and complex designs, identification of the appropriate level for tests and full reporting of outcomes |
| ☐ | ☒ | Estimates of effect sizes (e.g. Cohen's *d*, Pearson's *r*), indicating how they were calculated |

*Our web collection on statistics for biologists contains articles on many of the points above.*

## Software and code

Policy information about availability of computer code

| | |
|---|---|
| Data collection | N/A (this study re-used data) |
| Data analysis | As described and cited in the Methods section, the openly available software were:<br>R package 'aberrant' version 1.0<br>DeepVariant 0.10.0<br>plink v1.90b6.26<br>snpEff v5.1d<br>snpEff toolbox snpSift 5.1d<br>regenie v3.2.5<br>METAL (July 2010 version)<br>BHR 0.1.0<br>NMDEscPredictor https://nmdprediction.shinyapps.io/nmdescpredictor/ (no version number apparent at web interface)<br>MutationExplorer https://mutationexplorer.vda-group.de/mutation_explorer/ (no version number apparent at web interface)<br>blastp https://blast.ncbi.nlm.nih.gov/Blast.cgi?PAGE=Proteins (no version number apparent at web interface)<br>R software v4.2.1 |

For manuscripts utilizing custom algorithms or software that are central to the research but not yet described in published literature, software must be made available to editors and reviewers. We strongly encourage code deposition in a community repository (e.g. GitHub). See the Nature Portfolio guidelines for submitting code & software for further information.

## Data

Policy information about availability of data

All manuscripts must include a data availability statement. This statement should provide the following information, where applicable:

- Accession codes, unique identifiers, or web links for publicly available datasets
- A description of any restrictions on data availability
- For clinical datasets or third party data, please ensure that the statement adheres to our policy

The primary data used in this study are from the UK Biobank 29,90,91,93,94. The individual-level data can be provided by UK Biobank pending scientific review and a completed material transfer agreement. Requests for the data should be submitted to the UK Biobank: www.ukbiobank.ac.uk. UK Biobank data field/resource/category codes were: Handedness (chirality/laterality) (data-field 1707), 'Sex' (data-field 31), 'Year of birth' (data-field 34), 'Country of birth' (data-field 1647), 'Part of a multiple birth' (data-field 1777), genetic ancestry principal components (data-field 22009), self-reported ethnic identities (data-field 21000), 'Speech Reception Threshold' (fields 22219 (left ear) and 20021 (right ear)), 'Visual acuity' (fields 5187 (left eye) and 5185 (right eye)), 'Hearing difficulties/problems' (field 2247), 'Hearing aid user' (field 3393), 'Eye problems/disorders' (field 6148), 'Wears glasses or contact lenses' (field 2207), exome sequence target regions (resource 3803), exome sequence data (category 170), genotype array data (category 263).

Other data sources were:

Ensembl database (release 105): http://dec2021.archive.ensembl.org/index.html

Database for nonsynonymous functional prediction (dbNSFP) (version 4.3a) 97.

National Centre for Biotechnology Information https://www.ncbi.nlm.nih.gov/ reference sequence for human TUBB4B transcript NM_006088, and human proteins TUBB4B (NP_006079), TUBB (UQL51120), TUBB1 (NP_110400), TUBB2A (NP_001060.1), TUBB2B (NP_821080), TUBB3 (NP_006077), TUBB4A (NP_001276058), TUBB6 (AAI11375), TUBB8 (NP_817124). Also TUBB4B in Pan troglodytes (NP_006079), Macaca mulatta (NP_006079), Mus musculus (NP_006079), Rattus norvegicus (NP_006079), Bos taurus (NP_006079), Canis lupus familiaris (NP_006079), Gallus gallus (NP_006079) and Xenopus tropicalis (NP_006079).

Protein Data Bank https://www.wwpdb.org/ human TUBB4B model 'AF-P68371-F1-model_v4'.

Our meta-analyzed gene-based association summary statistics (shown in Figure 1) accompany this paper as a Source Data file.

## Research involving human participants, their data, or biological material

Policy information about studies with human participants or human data. See also policy information about sex, gender (identity/presentation), and sexual orientation and race, ethnicity and racism.

**Reporting on sex and gender**

We refer to sex as biological attribute. Sex was included as a covariate effect in the analyses. Individuals with discrepancies between their self-reported sex and their genetically determined sex were excluded from the analyses as this can indicate error in data entry.

**Reporting on race, ethnicity, or other socially relevant groupings**

The rate of left-handedness can vary from roughly 2% to 14% in different regions of the world, which is thought primarily to reflect enforced right-hand use in some cultures (3, 5, 6, 41). To avoid confounding our genetic association analysis, we defined four separate, genetically homogeneous groups of UK Biobank individuals that correspond to major world ancestries, using a combination of self-reported ethnicity and data-driven genetic clustering: Asian ancestry, Black ancestry, Chinese ancestry, and White ancestry (see Methods and Supplementary Figure 1). As expected, the rate of left-handedness varied between these clusters (Table 1). Within each cluster separately we would then test the association of genetic variants with handedness, and finally meta-analyze across clusters.

**Population characteristics**

We made use of the the UK Biobank adult population dataset. For all individuals with stable handedness data, we selected additional variables to use as covariates: 'Sex' (data-field 31), 'Year of birth' (data-field 34), 'Country of birth' (UK versus elsewhere; data-field 1647), 'Part of a multiple birth' (data-field 1777), the first 40 principal components derived from common variant genotype data that capture population ancestry (data-field 22009), and the exome sequencing batch (i.e. a binary variable to indicate whether an individual was sequenced as part of the first 50,000 exome release or subsequent releases, due to a difference in the flow cells used).

Pasted from Supplementary Table 11:

Excluded Included Excluded (%) Included (%)

Handedness Right 103689 313271 93.6% 87.5%

Left 5705 38043 5.2% 10.6%

Both hands equally 1361 6511 1.2% 1.8%

Sex Female 62635 191802 56.2% 53.6%

Male 48856 166023 43.8% 46.4%

Country of birth England 80295 284848 72.9% 79.6%

Wales 4322 16437 3.9% 4.6%

Scotland 7602 30196 6.9% 8.4%

Northern Ireland 527 2360 0.5% 0.7%

Republic of Ireland 1006 3621 0.9% 1.0%

Elsewhere 16464 20363 14.9% 5.7%

Ancestry cluster White 68197 343781 61.2% 96.1%

Asian 845 7052 0.8% 2.0%

Black 579 5729 0.5% 1.6%

Chinese 96 1263 0.1% 0.4%

Not clustered / mixed cluster 41774 0 37.5% 0.0%

Part of multiple birth No 100980 350039 97.7% 97.8%

Yes 2400 7786 2.3% 2.2%
Exome sequencing batch First 50k 11362 38310 10.2% 10.7%
All other 100129 319515 89.8% 89.3%
Year of Birth (Mean, SD) - 1952 (8) 1951 (8) - -

Recruitment

We made use of the the UK Biobank adult population dataset: https://www.ukbiobank.ac.uk/

Ethics oversight

For this study we used data from the UK Biobank 90, 91. The UK Biobank received ethical approval from the National Research Ethics Service Committee North West-Haydock (reference 11/NW/0382), and all of their procedures were performed in accordance with the World Medical Association guidelines 29. Written informed consent was provided by all of the enrolled participants.
The present study was conducted as part of UK Biobank registered project 16066, with Clyde Francks as the principal investigator. The study design and conduct complied with all relevant regulations regarding the use of human study participants and was conducted in accordance to the criteria set by the Declaration of Helsinki, with approval from the Ethics Committee Faculty of Social Sciences, Radboud University Nijmegen.

Note that full information on the approval of the study protocol must also be provided in the manuscript.

# Field-specific reporting

Please select the one below that is the best fit for your research. If you are not sure, read the appropriate sections before making your selection.

- [x] Life sciences
- [ ] Behavioural & social sciences
- [ ] Ecological, evolutionary & environmental sciences

For a reference copy of the document with all sections, see nature.com/documents/nr-reporting-summary-flat.pdf

# Life sciences study design

All studies must disclose on these points even when the disclosure is negative.

Sample size

No statistical method was used to predetermine the sample size. It could not be estimated in advance what type of genetic effects on handedness due to rare protein-coding variants would be present in the data, as no study has previously addressed this question. We therefore used the maximum available sample size to maximise the available statistical power to screen the genome for such effects.
The sample therefore included all participants from the UK Biobank who met a set of criteria according to data availability, consistency, and population genetic ancestry, as detailed in the Methods section and repeated here below. The multi-step process through which we arrived at the study's final sample size is most correctly and accurately described by pasting from the Methods section, because all of this information determined the final sample size:

Phenotype data were obtained from data release version 10.1 (available on the research analysis platform since 14 April 2022), and the whole exome sequence data were from release version 12.1 (available on the platform since 29 June 2022). We selected 'Handedness (chirality/laterality)' (data-field 1707) as our primary phenotype, which was self-reported according to the question 'Are you right or left handed?' (presented on a touchscreen). Possible answers were 'right-handed', 'left-handed', 'use both left- and right hands equally' and 'prefer not to answer'. The latter was treated as missing data. Answers were recorded at a maximum of three visits to a UK Biobank assessment center. We used the handedness reported at the first non-missing instance. For individuals who had reported their hand preference at multiple instances, those who were inconsistent in their reported handedness were excluded.
For all individuals with stable handedness data, we selected additional variables to use as covariates: 'Sex' (data-field 31), 'Year of birth' (data-field 34), 'Country of birth' (data-field 1647), 'Part of a multiple birth' (data-field 1777), the first 40 principal components derived from common variant genotype data that capture population ancestry (data-field 22009), and the exome sequencing batch (i.e. a binary variable to indicate whether an individual was sequenced as part of the first 50,000 exome release or subsequent releases, due to a difference in the flow cells used). 'Country of birth' and 'Part of a multiple birth' could be recorded at multiple instances, and again we set these to missing if individuals reported inconsistent answers.
Defining ancestry clusters
We first grouped 469,804 individuals with exome data into five ancestry groups according to self-reported ethnic identities in UK Biobank data-field 21000:
- Asian or Asian British (includes sub-fields Indian, Pakistani, Bangladeshi and 'any other Asian background').
- Black or Black British (includes sub-fields Caribbean, African, and 'any other Black background').
- Chinese (includes only Chinese background).
- White (includes British, Irish, and 'any other white background').
- Mixed
Answers of 'Do not know', 'Prefer not to answer', or 'Other' were set to missing. Ethnicity was reported at up to four visits. Individuals were only assigned to one of the five ancestry groups if they had non-missing data for at least one instance, and consistently reported their ethnicity with respect to these five groups if reported at multiple instances. For each of the five self-reported ethnic groups separately, we then applied a Bayesian clustering algorithm in the R package 'aberrant' version 1.0 92 to genetic ancestry principal components 1-6 (from data-field 22009). This software seeks to define clusters of datapoints and any outliers from them. The 'aberrant' package can only cluster along two dimensions, and was therefore run separately three times for each self-reported ethnic grouping: first on principal components 1&2, then 3&4, then 5&6, with inlier threshold lambda=40. Individuals in the intersect of all three clusters for a given ethnicity were then assigned to one final genetically-informed cluster for each ethnic group.
For the 'mixed' ancestry group we obtained a highly dispersed cluster, and therefore these individuals were excluded. See Table 1, Supplementary Figure 1 and Supplementary Table 1 for further information.
Sample-level filtering

There were initially 469,316 individuals with whole exome sequence data, and who consistently reported their handedness, country of birth and whether they were part of a multiple birth. We then applied further individual-level quality control. First, individuals with missing data for one or more covariates defined above were excluded. Then we excluded individuals with discordant self-reported and genetically determined sex, as well as those not included in one of the genetically-informed ancestry clusters as described above. For pairs of related individuals inferred as third-degree relatives or closer (kinship coefficient > 0.0442) based on common variant data 90, we excluded one individual from each pair, prioritizing the removal of right-handed individuals and those present in multiple pairs, but otherwise randomly.

In total, 111,491 individuals were removed by all of these steps together, which left 357,825 remaining individuals. Supplementary Table 1 shows that the majority of exclusions occurred for one of two reasons:

1. 39,170 individuals fell outside of all four of the genetically-informed ancestry clusters that were retained: Asian or Asian British, Black or Black British, Chinese, or White. As the rate of left-handedness varied with ancestry (Table 1), then the excluded sample was expected to differ from the included sample in terms of handedness, and other demographic features that correlate with handedness (see details in Supplementary Table 11).

2. 62,882 individuals were excluded due to being related to another individual at third degree level or higher. As mentioned above, when such a pair or relatives comprised one right-handed and one-left-handed individual, the left-handed individual was retained. This was done to maximise the number of left-handers for statistical power in genetic association analysis. Again, this meant that the excluded sample necessarily differed from the included sample in terms of handedness, and other demographic features that correlate with handedness (see details in Supplementary Table 11).

Of the remaining 357,825 individuals after sample-level filtering, 313,271 were right-handed, 38,043 were left-handed, and 6,511 reported using both hands equally. See Table 1 for a breakdown by ancestry clusters. As mentioned earlier, the 'both hands equally' phenotype was not considered in our genetic association and heritability analyses due to a relatively low sample size and poor repeatability, but these individuals were included in our exome sequence pre-processing pipeline.

Data exclusions

The exclusion criteria were pre-established. There were multiple exclusion criteria based on the availability and consistency of the different types of data, as well as population genetic homogeneity. The exclusion process is most correctly and accurately described by pasting from the Methods section, as all of these steps are relevant to which data were excluded:

Phenotype data were obtained from data release version 10.1 (available on the research analysis platform since 14 April 2022), and the whole exome sequence data were from release version 12.1 (available on the platform since 29 June 2022). We selected 'Handedness (chirality/laterality)' (data-field 1707) as our primary phenotype, which was self-reported according to the question 'Are you right or left handed?' (presented on a touchscreen). Possible answers were 'right-handed', 'left-handed', 'use both left- and right hands equally' and 'prefer not to answer'. The latter was treated as missing data. Answers were recorded at a maximum of three visits to a UK Biobank assessment center. We used the handedness reported at the first non-missing instance. For individuals who had reported their hand preference at multiple instances, those who were inconsistent in their reported handedness were excluded.

For all individuals with stable handedness data, we selected additional variables to use as covariates: 'Sex' (data-field 31), 'Year of birth' (data-field 34), 'Country of birth' (data-field 1647), 'Part of a multiple birth' (data-field 1777), the first 40 principal components derived from common variant genotype data that capture population ancestry (data-field 22009), and the exome sequencing batch (i.e. a binary variable to indicate whether an individual was sequenced as part of the first 50,000 exome release or subsequent releases, due to a difference in the flow cells used). 'Country of birth' and 'Part of a multiple birth' could be recorded at multiple instances, and again we set these to missing if individuals reported inconsistent answers.

Defining ancestry clusters

We first grouped 469,804 individuals with exome data into five ancestry groups according to self-reported ethnic identities in UK Biobank data-field 21000:

- Asian or Asian British (includes sub-fields Indian, Pakistani, Bangladeshi and 'any other Asian background').
- Black or Black British (includes sub-fields Caribbean, African, and 'any other Black background').
- Chinese (includes only Chinese background).
- White (includes British, Irish, and 'any other white background').
- Mixed

Answers of 'Do not know', 'Prefer not to answer', or 'Other' were set to missing. Ethnicity was reported at up to four visits. Individuals were only assigned to one of the five ancestry groups if they had non-missing data for at least one instance, and consistently reported their ethnicity with respect to these five groups if reported at multiple instances. For each of the five self-reported ethnic groups separately, we then applied a Bayesian clustering algorithm in the R package 'aberrant' version 1.0 92 to genetic ancestry principal components 1-6 (from data-field 22009). This software seeks to define clusters of datapoints and any outliers from them. The 'aberrant' package can only cluster along two dimensions, and was therefore run separately three times for each self-reported ethnic grouping: first on principal components 1&2, then 3&4, then 5&6, with inlier threshold lambda=40. Individuals in the intersect of all three clusters for a given ethnicity were then assigned to one final genetically-informed cluster for each ethnic group.

For the 'mixed' ancestry group we obtained a highly dispersed cluster, and therefore these individuals were excluded. See Table 1, Supplementary Figure 1 and Supplementary Table 1 for further information.

Sample-level filtering

There were initially 469,316 individuals with whole exome sequence data, and who consistently reported their handedness, country of birth and whether they were part of a multiple birth. We then applied further individual-level quality control. First, individuals with missing data for one or more covariates defined above were excluded. Then we excluded individuals with discordant self-reported and genetically determined sex, as well as those not included in one of the genetically-informed ancestry clusters as described above. For pairs of related individuals inferred as third-degree relatives or closer (kinship coefficient > 0.0442) based on common variant data 90, we excluded one individual from each pair, prioritizing the removal of right-handed individuals and those present in multiple pairs, but otherwise randomly.

In total, 111,491 individuals were removed by all of these steps together, which left 357,825 remaining individuals. Supplementary Table 1 shows that the majority of exclusions occurred for one of two reasons:

1. 39,170 individuals fell outside of all four of the genetically-informed ancestry clusters that were retained: Asian or Asian British, Black or Black British, Chinese, or White. As the rate of left-handedness varied with ancestry (Table 1), then the excluded sample was expected to differ from the included sample in terms of handedness, and other demographic features that correlate with handedness (see details in Supplementary Table 11).

| | |
|---|---|
| | 2. 62,882 individuals were excluded due to being related to another individual at third degree level or higher. As mentioned above, when such a pair or relatives comprised one right-handed and one-left-handed individual, the left-handed individual was retained. This was done to maximise the number of left-handers for statistical power in genetic association analysis. Again, this meant that the excluded sample necessarily differed from the included sample in terms of handedness, and other demographic features that correlate with handedness (see details in Supplementary Table 11).<br>Of the remaining 357,825 individuals after sample-level filtering, 313,271 were right-handed, 38,043 were left-handed, and 6,511 reported using both hands equally. See Table 1 for a breakdown by ancestry clusters. As mentioned earlier, the 'both hands equally' phenotype was not considered in our genetic association and heritability analyses due to a relatively low sample size and poor repeatability |
| Replication | We are not aware of a suitable replication sample to match the scale of the UK Biobank. Tens of thousands of indiivduals with handedness and exome sequence data would be required. |
| Randomization | This was not a feature of our observational study. |
| Blinding | This was not a feature of our observational study. |

# Reporting for specific materials, systems and methods

We require information from authors about some types of materials, experimental systems and methods used in many studies. Here, indicate whether each material, system or method listed is relevant to your study. If you are not sure if a list item applies to your research, read the appropriate section before selecting a response.

## Materials & experimental systems

| n/a | Involved in the study |
|---|---|
| ☒ | ☐ Antibodies |
| ☒ | ☐ Eukaryotic cell lines |
| ☒ | ☐ Palaeontology and archaeology |
| ☒ | ☐ Animals and other organisms |
| ☐ | ☒ Clinical data |
| ☒ | ☐ Dual use research of concern |
| ☒ | ☐ Plants |

## Methods

| n/a | Involved in the study |
|---|---|
| ☒ | ☐ ChIP-seq |
| ☒ | ☐ Flow cytometry |
| ☒ | ☐ MRI-based neuroimaging |

## Clinical data

Policy information about clinical studies

All manuscripts should comply with the ICMJEguidelines for publication of clinical research and a completedCONSORT checklist must be included with all submissions.

| | |
|---|---|
| Clinical trial registration | We made use of the the UK Biobank adult population dataset, including some of its clinical data (related to vision and hearing loss). This was an observational study and not a clinical trial. |
| Study protocol | We made use of the the UK Biobank adult population dataset, including some of its clinical data (related to vision and hearing loss). This was an observational study and not a clinical trial. |
| Data collection | We made use of the the UK Biobank adult population dataset: https://www.ukbiobank.ac.uk/ |
| Outcomes | This was an observational study and not a clinical trial. |
